# Self-Discharge Processes in Symmetrical Supercapacitors with Activated Carbon Electrodes

**DOI:** 10.3390/ma16196415

**Published:** 2023-09-26

**Authors:** Alexey Yu. Rychagov, Valentin E. Sosenkin, Marianna Yu. Izmailova, Evgeny N. Kabachkov, Yury M. Shulga, Yury M. Volfkovich, Gennady L. Gutsev

**Affiliations:** 1 A.N. Frumkin Institute of Physical Chemistry and Electrochemistry, Russian Academy of Sciences, Leninsky Pr. 31, 119071 Moscow, Russia; rychagov69@mail.ru (A.Y.R.); vsosenkin@mail.ru (V.E.S.); maryann21@rambler.ru (M.Y.I.); yuvolf40@mail.ru (Y.M.V.); 2 Federal Research Center of Problem of Chemical Physics and Medicinal Chemistry, Russian Academy of Sciences, Chernogolovka, 142432 Moscow, Russia; en.kabachkov@gmail.com (E.N.K.); yshulga@gmail.com (Y.M.S.); 3 Department of Physics, Florida A&M University, Tallahassee, FL 32307, USA

**Keywords:** self-discharge, electric double-layer supercapacitor, activated carbon electrode

## Abstract

The self-discharge of an electric double-layer capacitor with composite activated carbon electrodes and aqueous electrolyte (1 M MgSO_4_) was studied in detail. Under a long-term potentiostatic charge (stabilization), a decrease in the discharge capacity was observed in the region of voltages exceeding 0.8 V. The self-discharge process consists of two phases. In the initial phase, the cell voltage drop is due to the charge redistribution inside electrodes. During the main phase, the charge transfer between the electrodes determines the voltage drop. The optimal stabilization time of the self-discharge was found to be 50 min at 1.4 V. Hydrophilization of the negative electrode occurred during long-term polarization due to the formation of epoxy functional groups.

## 1. Introduction

In recent years, many studies have been performed on supercapacitors (SC) and much progress has been made [1,2]. Supercapacitors have many advantages over batteries: nearly infinite cycle life, much higher power density, and many charge–discharge cycles. In addition, they are inexpensive and eco-friendly. There is a possibility of achieving up to 100% energy storage efficiency for double-layer capacitors (DSCs) due to the lack of polarization of electrode reactions. The main disadvantage of supercapacitors is their significant self-discharge. The following self-discharge mechanisms have been found [3]:

1. Ionic mass transfer through the pores along the thickness of the electrode [4,5,6,7,8]. The voltage drops after preliminary long-term charging (for at least 4 h) is significantly reduced, and the precharge process allows the ions to be more evenly distributed over the electrode surface pores.
2. Carbon oxidation. References [7,8] show that self-discharge depends on the state of the surface of activated carbon. Activated carbon (AC) shows a 50% reduction in self-discharge when the surface is completely oxidized as a result of electrochemical cycling compared to the initial state.
3. Ionic transfer [1,9,10,11,12,13,14]. Products of redox reactions can diffuse from a negative electrode to a positive electrode and vice versa: for example, due to the presence of iron ions in the electrolyte solution.

The following limiting stages of ion transfer between electrodes can be distinguished:

  (a) The stage of polarization of electrodes due to reactions on their surfaces, which depends on the surface catalytic capability;
  (b) The barrier stage in the formation of the of electric double layer (EDL);
  (c) The stages of diffusion and ohmic transfer of ions between electrodes.

4. Ion transfer from places with higher concentrations to places with lower concentrations [15,16,17,18,19,20,21,22,23,24].

The main goal of this work is to study the self-discharge of symmetric supercapacitors with composite electrodes based on Norit activated carbon (Netherlands). The objectives of this study also include the determination of the minimal and optimal cell exposure time at the maximum charge voltage that is required to equalize the potential in the porous structure of an electrode; the evaluation of the leading mechanisms of self-discharge at different time intervals; and the dependence of compositions of functional surface groups on the electrode polarity.

The porous structure and hydrophilic–hydrophobic properties of Norit AC and electrodes based on AC were studied in a wide range of pore radii, from tenths of a nanometer to one-hundred micrometers [3], using the method of standard contact porosimetry. It was found that studied objects had both hydrophilic and hydrophobic pores, and that the proportion of hydrophobic pores was greater in electrodes due to the presence of polytetrafluoroethylene and carbon black. It was shown that the main contribution to the specific surface area was due to micropores with radii less than 1 nm.

## 2. Experimental Section

### 2.1. Electrode Fabrication

Our electrodes were prepared by mixing activated carbon Norit DLC Supra 30 (Cabot Norit Netherlands, Klazienaveen, The Netherlands) with an electrically conductive filler (technical carbon UM-76) in a ratio of 5:1, and isopropanol was gradually added until a homogeneous suspension was formed. To the obtained mixture, an aqueous suspension of polytetrafluoroethylene (a binder) in an amount of 4% (in terms of dry components) was added and stirred vigorously. The weight ratio of the dry carbon component and isopropyl alcohol in the suspension was 1:6. The resulting quaternary suspension was filtered to remove excess alcohol. After filtration, the mixture was rolled to the required thickness and dried under vacuum to obtain compact dry electrodes.

### 2.2. Electrochemical Measurements

Electrochemical studies were carried out in a two-electrode cell where two electrodes based on activated carbon were separated by a separator, 160 µm thick highly porous (60–80%) nonwoven PP cloth (FS 2226, Velidon, Germany). The mass and area of one electrode were 40 mg and 3 cm^2^, respectively. The electrochemical part was compressed under a pressure of 1 kg/cm^2^ between two non-porous graphite washers acting as current collectors. For the electrolyte, 1 M MgSO_4_ was used. The electrolytes were prepared with 18 MΩ cm^−1^ water (prepared with Millipore Direct-Q 3 UV), puriss grade, from Aldrich. Before assembling the cell, three electrodes and a separator were kept in excess electrolyte for 5 days. During the entire electrochemical study, the control electrode was immersed in the electrolyte. At the end of each experiment, the positive, negative, and control electrodes were washed separately. After washing and drying, the electrodes were transferred for physical and chemical studies.

Galvanostatic, impedance, and voltametric studies were carried out using a P-40X potentiostat–galvanostat with a FRA-24M impedance measurement module (Electrochemical instruments, Chernogolovka, Russia). 

### 2.3. Contact Angle Measurement

The measurement of contact angle of wetting (θc) of a flat surface with water and the drop contour analysis were performed on an OSA 20 instrument (DataPhysics Instruments GmbH, Filderstadt, Germany) at room temperature. The results were processed according to the Young–Laplace method.

### 2.4. IR Spectroscopy

The IR spectra were obtained using a Perkin-Elmer Spectrum Two FTIR spectrometer (PerkinElmer Inc., Waltham, MA, USA) within the mid-IR (4000–450 cm^−1^) range with an ATR attachment. Thirty-two scans were recorded at room temperature with a resolution of 1 cm^−1^.

### 2.5. X-ray Photoelectron Spectroscopy

The XPS measurements were performed on an X-ray photoelectron spectrometer (SPECS PHOIBOS 150 MCD9 GmbH). The spectra were obtained using Mg Kα radiation (hν = 1253.6 eV) at a fixed analyzer pass energy (40 eV for survey spectra and 10 eV for individual lines). The vacuum in the spectrometer chamber did not exceed 4 × 10^−8^ Pa. For elemental analysis, a survey spectrum was recorded with the binding energy (E_b_) range extending from zero to >1000 eV in 1.00 eV increments, and the spectra of individual lines were recorded in 0.03 eV increments.

## 3. Results and Discussion

### 3.1. Electrochemical Measurements

**Figure 1:**
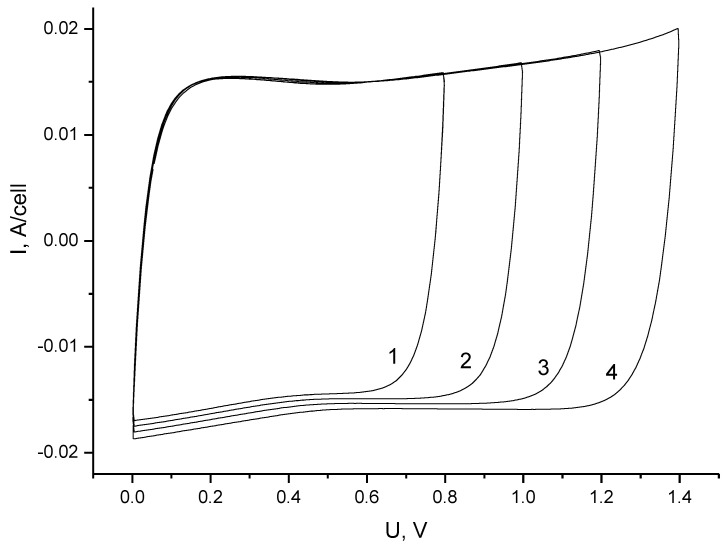
Cyclic voltammograms of symmetrical supercapacitors at a scan rate of 10 mV/s. Curve 1 is for ΔU = 0.8 V, curve 2 is 1.0 V, curve 3 is for 1.2 V, and curve 4 is for 1.4 V.

The assembled symmetrical supercapacitor cell was subjected to preliminary cyclic tests for charge–discharge stability. Since it was shown in [15] that the cells can be charged up to a voltage of 1.4 V (for an electrolyte of 1 M MgSO_4_), this value was accepted as the maximum allowable charge voltage. The cyclic voltammetry (CV) curves (Figure 1) have a minimum at about 0.6 V when charging the cell at voltages exceeding 1 V. Probably, such a minimum appears as a consequence of the shift in potential of the zero-charge toward polarization of each of the electrodes. The minimum, as a rule, is maintained at zero voltage when changing polarity. The shift in potential of the zero-charge is characteristic of pH-neutral aqueous electrolytes and is primarily associated with redox properties of surface functional groups of activated carbons. It seems that this is related to the fact that the charging of an electric double layer (EDL) at a low voltage is determined by ions of the same charge as the electrode polarity [25,26,27].

**Figure 2:**
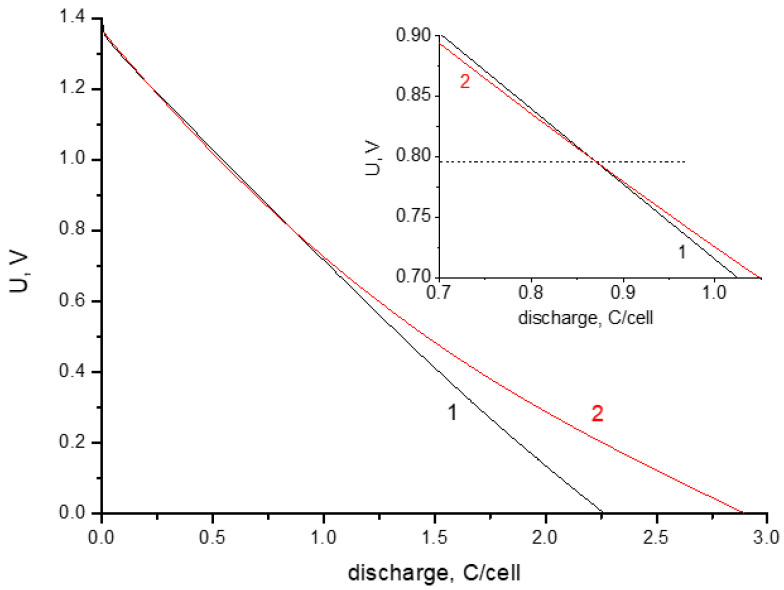
Galvanostatic discharge curves at 10 mA: 1—without stabilization; 2—with stabilization for 3 h at 1.4 V. The inset shows an enlarged portion of the curves in the vicinity of their intersection.

The permissible voltage of a capacitor is determined by the reversibility regions of the charge–discharge processes. Its value depends on the type of electrolyte and the resistance of electrodes toward oxidation (for aqueous electrolytes). At charge voltages up to 0.9 V, the main current-forming processes are the EDL charging and redox reactions of functional groups with high exchange currents. At voltages exceeding 0.9 V, the charging processes are becoming less reversible because of contributions of the specific adsorption of ions on the surface of activated carbon. The presence of slightly reversible processes is especially noticeable during prolonged exposures of supercapacitor cells to high voltages and leads to an increase in discharge capacity at low voltages (Figure 2).

Figure 2 shows the dependence of voltage on discharge capacity. Capacity is calculated by multiplying the discharge time by the discharge current. The discharge current is 10 mA per cell or 250 mA/g per electrode. The average capacitance corresponding to discharge curve 1 (Figure 2) is close to 2 F per cell or about 100 F/g with respect to one electrode. An increase in the discharge capacity obtained as a result of stabilization is observed only in the region of low voltages (Figure 2, curve 2), while the capacity in the region of voltages above 0.8 V turns out to be somewhat smaller than the capacity for the discharge without stabilization. The difference between amounts of charge during long-term stabilization and the discharge (Coulomb efficiency) is determined not only by the supercapacitor self-discharge, but also by chemisorption processes. The desorption of particles (ions or radicals) strongly bound to the carbon surface requires significant discharge overvoltage and cannot be fully realized, even with prolonged short circuiting. This is indirectly indicated by the observation that the total increase in the discharge energy obtained due to stabilization is insignificant (11%), whereas the discharge capacity near 0 V increases by 54% (up to 3.2 F). The process of chemical interactions of the electrolyte with the carbon electrode is substantially slowed and leads to neither a noticeable increase in the characteristics nor significant degradation of the cell. However, as mentioned above, keeping the cell at the maximum charge voltage makes it possible to reduce the self-discharge caused by the uneven distribution of charges in the electrode porous structure. Hence, it follows that holding the supercapacitor cell at the maximum charge voltage for some time should allow attainment of the optimal value for reducing the self-discharge and to limit the chemisorption interaction between the electrolyte and electrodes.

**Figure 3:**
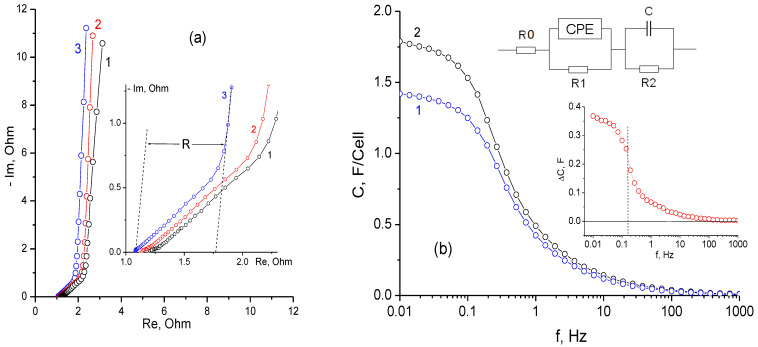
(**a**) The Nyquist diagram for different supercapacitor cell voltages: 1.4 V (1), 0.8 V (2), and 0.0 V (3) measured in the frequency range from 100 kHz to 0.01 Hz. The inset shows the intermediate frequency region. (**b**) Dependence of the capacitance on the frequency at a voltage of 0 V: 1 corresponds to the original cell; and 2 corresponds to the cell after a long exposure at the maximum charge voltage. The inserts show a difference curve and an equivalent electrical circuit.

Despite an increase in capacitance during discharge (Figure 2), the reversible differential capacitance determined by impedance measurements shows a slight decrease in the low-frequency region (Figure 3a).

The impedance spectra of a supercapacitor cell obtained at different voltages immediately after stabilization cycling are shown in Figure 3а. The impedance was measured from high to low voltage. The most important characteristic of a symmetrical supercapacitor impedance is the value of resistance for the transition from the polarization (the slope is 45°) to the capacitive part (the slope is close to 90°) [28]. This resistance R (see the inset in Figure 5) for a real supercapacitor usually determines the effective ionic conductivity, which depends on the porous structure and thickness of electrodes. According to the impedance measurements, the resistance value R in the discharged state is lower than that for the charged cell. This may be due to the influence of the self-discharge current (as the movement of particles could prevent the free migration of ions) on the measured ionic conductivity in the electrode pores. The capacitance calculated from impedance measurements using an equivalent circuit (Figure 3b) is close to the capacitance calculated from galvanostatic curves (Figure 2).

**Figure 4:**
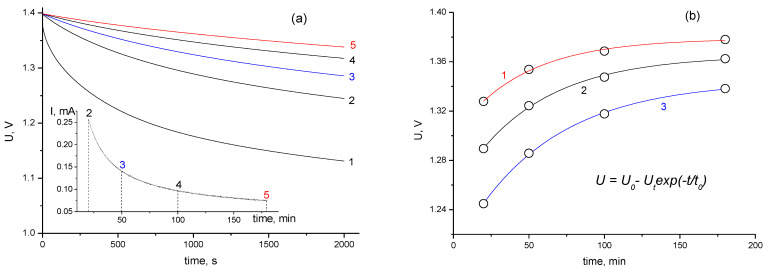
The self-discharge process of a supercapacitor cell. (**а**) Different stabilization times: without stabilization (1), for 20 min (2), for 50 min (3), for 100 min (4), and for 180 min (5). The inset shows the current drop during stabilization. (**b**) An approximation of the dependence of voltage on stabilization time at different discharge times: for 500 s (1), for 1000 s (2), and for 2000 s (3).

A series of experiments was carried out to study the effects of stabilization time on self-discharge at 1.4 V. The cell was charged with a constant current of 10 mA to a voltage of 1.4 V, followed by holding the limiting charge voltage from 0 to 180 min. When charging was finished, the cell was left for 20-h self-discharge and then discharged with a current of 10 mA. Figure 4a shows the voltage drop (within 2000 s) obtained after various cell stabilization times at a voltage of 1.4 V. As can be seen from Figure 3a, the voltage drop rate in the initial self-discharge period is strongly dependent on the stabilization time. However, after a relatively short self-discharge time of 2000 s, the voltage drop rates began to equalize and approach a value of about 1.5 mV/min. In the first approximation, the voltage drop rate is proportional to the current corresponding to the cell stabilization time (Figure 4a, inset).

The dependence of the voltage loss on the stabilization time for the initial stage of self-discharge is shown in Figure 4b. For the proposed model, the voltage can be described as U = U_0_ − U_t_ *exp*(−t/t_0_), where U_0_ is the cell voltage at an infinite stabilization time, t_0_ is the effective stabilization time (an incubation period), and U_t_ is the voltage loss at the effective stabilization time. In line with their physical meaning, parameters U_0_ and U_t_ depend on the self-discharge time, whereas t_0_ must be constant, depending only on the charge voltage and structural–capacitive characteristics of the electrode. Under the experimental conditions, the parameter t_0_ shows a slight increase with an increase in the self-discharge time. For the curves shown in Figure 3b, its value increases from 46 min (curve 1) to 58 min (curve 3), which can be explained by the simplified type of the model and the drop in the EDL capacity observed during long-term stabilization. For the initial stage of self-discharge, it is characteristic that processes of charge redistribution in the electrodes have the greatest influence on the voltage drop.

For each stabilization time, the cell was disconnected from the measuring device and left to self-discharge at room temperature. After 20 h, the residual voltage was measured and the discharge galvanostatic curve was recorded with a current of 10 mA.

**Figure 5:**
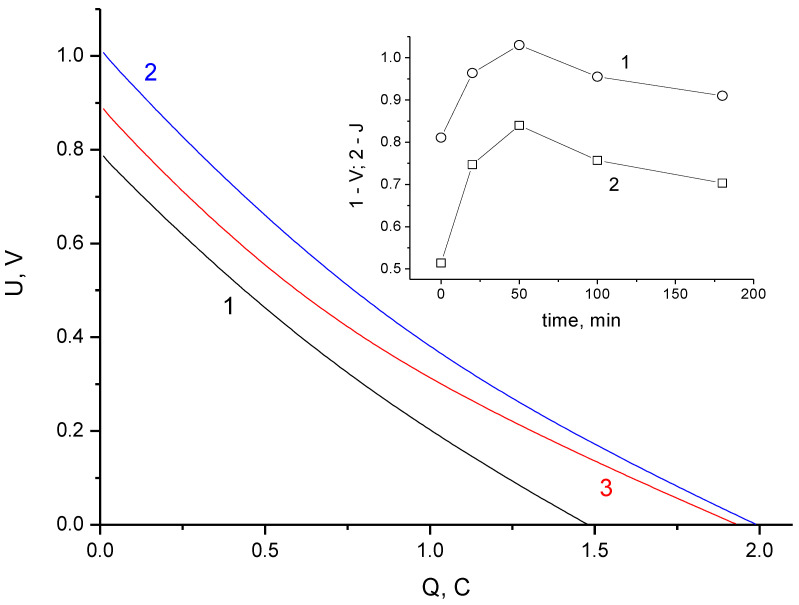
Galvanostatic discharge curves at 10 mA obtained after 20 h self-discharge at different stabilization times: without stabilization (1), 20 min (2), and 50 min (3). The inset shows the cell energy vs stabilization time.

The discharge curves obtained after a 20-h self-discharge period are shown in Figure 5. In contrast to the initial stage of self-discharge, the residual voltage of a 20 h self-discharge has a clearly defined maximum, shown in the insert of Figure 4. The shape of discharge curve for a cell charged during long-term stabilization shows an increase in capacitance at the end of discharge (up to 3.2 F), as well as a cell without self-discharge. The insert in Figure 4 shows the dependence of the residual voltage and energy on the stabilization time during charging. The voltage loss at the maximum (50 min stabilization) for 20 h at a temperature of 25 °C was 26%; the energy loss was 44%, whereas the energy loss was 57% for the stabilization time of 180 min. The average self-discharge current for 20 h can be obtained by dividing the loss of electricity (in C) by the time (in s). The difference between the values of the discharge amount of electricity presented in Figure 2 and Figure 5 shows that the average self-discharge current with 50 min stabilization (11 μA) is lower than the average current with long-term stabilization (14 μA). Considering that for the initial stage (Figure 4), the self-discharge current for a cell without stabilization exceeds the current of the stabilized cell, one can argue that there is a change in the initial self-discharge process during the first hours. The optimal stabilization time (50 min) obtained during long-term self-discharges turned out to be close to the parameter t_0_ value, calculated by approximating the initial stage of self-discharge (Figure 4b). 

**Figure 6:**
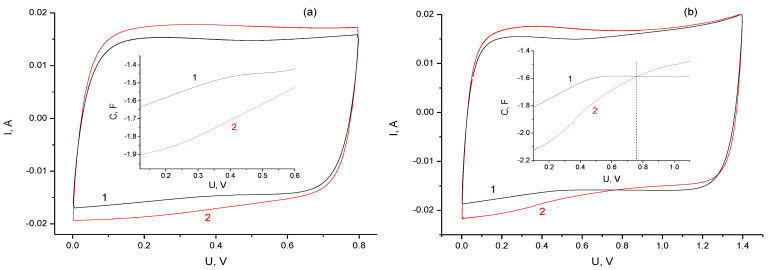
The CV curves at a scan rate of 10 mV/s: curve 1 is obtained for a freshly assembled cell; curve 2 is obtained for the cell used in our self-discharge studies. The range up to 0.8 V (**a**) and the range up to 1.4 V (**b**). The inserts compare the discharge capacitance characteristics.

To understand the nature of an increase in the self-discharge rate at long stabilization times, the CV of a freshly assembled cell was compared with the CV of the cell after being used in experiments, as shown in Figure 6.

As can be seen in Figure 6a, the main change in the CV shape in the region of high reversibility (up to 0.8 V) is associated with an increase in currents due to an increase in the capacitance of Faraday processes. Functional groups formed on the surface as a result of oxidation partially block the surface, which leads to a decrease in the capacity of the EDL in the high-voltage region. An increase in capacitance in the low-voltage region (less than 0.8 V) can be considered as a reversible reaction of the addition of hydrogen to carbonyls similar to the well-known quinone–hydroquinone reaction (˃С=О + Н^+^ + е^−^ = ˃С-О-Н). 

The CVs of a freshly assembled cell and a cell after testing over the full cycling range (up to 1.4 V) are compared in Figure 6b. As can be seen, the cathodic part of the curve for the cell that was under test before is divided into two regions with a large spread (25%) of the measured capacitances (Figure 6b, inset). Apparently, the capacitance of the region above 0.8 V is determined only by the EDL, for which the value is limited due to the blocking of the electrode surface by functional groups and adsorbed particles. Taking into account the decrease in capacitance during long-term stabilization (Figure 2, inset), the loss of capacitance for the voltage region above 0.8 V can lead to an acceleration of the voltage drop during self-discharge. However, this mechanism of accelerated self-discharge at voltages above 0.8 V does not explain the appearance of an optimum in terms of residual charge. As shown above, the average self-discharge current increases with increasing stabilization time, which can be explained by the accumulation of charge carriers capable of being oxidized or reduced on counter electrodes, accelerating self-discharge. The charge carriers can be electrically neutral radicals (possible products of the water degradation), partially adsorbed on electrode surfaces. Under such conditions, the optimal stabilization time will be determined by the rate of saturation of surface vacancies with adsorbed radicals; that is, it will depend on the voltage and the nature of the material the electrodes are made of.

### 3.2. Contact Angle Measurements

The contact wetting angle (CWA) was measured for the electrodes under study. The original electrode, which was not subjected to polarization, showed significant hydrophobicity with a CWA exceeding 90° (Figure S1, Supporting Information). A decrease in the CWA to below 50° was observed (Figure S2, Supporting Information) for the negative electrode, which was subjected to reductive polarization, resulting in hydrophilization of electrode. For the positive electrode, which was at a high oxidation potential for a long time, a drop of water disappeared from the electrode surface within a few seconds (Video S1, Supporting Information). Thus, the positive electrode was the most hydrophilic. 

### 3.3. IR Spectroscop

**Figure 7:**
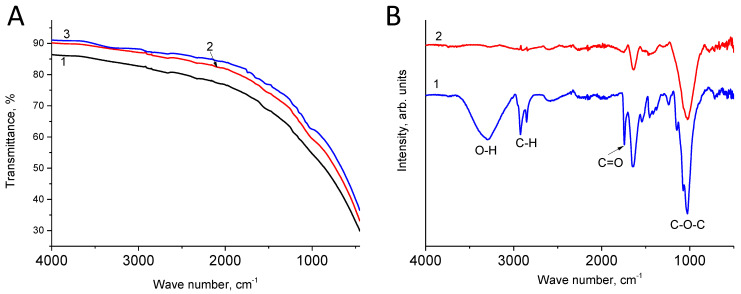
(**A**) The FTIR spectra of the initial electrode (1), the negative electrode (2) and the positive electrode (3); (**B**) the subtracted FTIR spectra: the spectrum of the initial electrode minus the spectrum of the positive electrode (1); the spectrum of the initial electrode minus the spectrum of the negative electrode (2).

The FTIR spectra of our electrodes are shown in the inset of Figure 7A. Due to rather high conductivity, we did not observe pronounced features in the spectra. However, when subtracting the spectrum, distinct peaks cannot be observed (Figure 7B). These peaks correspond to the oxidized surface of the carbon material. Note that the peaks for the positive electrode are more intense. From this, we can conclude that the positive electrode is more oxidized compared to the negative electrode.

According to the literature data, wide bands in the range of 3620–3030 cm^−1^ and a maximum at 3296 cm^−1^ are usually related to O–H stretching vibrations [29,30,31]. Peaks at 2920 cm^−1^ and 2859 cm^−1^ are associated with the stretching vibrations of C–H bonds. These bands can be seen only for the positive electrode, which was not reduced under the experimental conditions. Therefore, their appearance cannot be directly related to electrochemical processes. The peak at 1740 cm^−1^ is due to vibration of the C=O bonds in carbonyl groups and/or ketones, which appear because of the electrochemical oxidation of carbon. Contributions to the intensity of the peak at 1645 cm^−1^ arise from both vibrations of double bonds C=C [32] and bending vibrations of water molecules. The most intense peaks for the positive and negative electrodes are observed at 1070 cm^−1^ and 1030 cm^−1^. According to [33], these peaks are caused by vibrations of the C–O–C bonds of epoxy functional groups.

### 3.4. X-ray Photoelectron Spectroscopy

The states of atoms in the near-surface layers of electrodes were analyzed by the XPS method. It can be seen in Table 1 that these near-surface layers contain nitrogen, fluorine, and sulfur in addition to carbon and oxygen. The presence of sulfur can be attributed to residual content of the electrolyte on the electrodes. Nitrogen can be incorporated into the electrode following its activation with nitric acid [34,35,36]. Fluorine incorporates into the electrodes during the electrode formation (see Section 2), because of the presence of a binder in the electrodes. As can be seen in Table 1, the electrode testing is accompanied by the oxidation of both electrodes; correspondingly, the carbon concentration decreases and the oxygen concentration increases. It can also be seen that the oxidation process proceeds more deeply in the case of the positive electrode.

**Figure 8:**
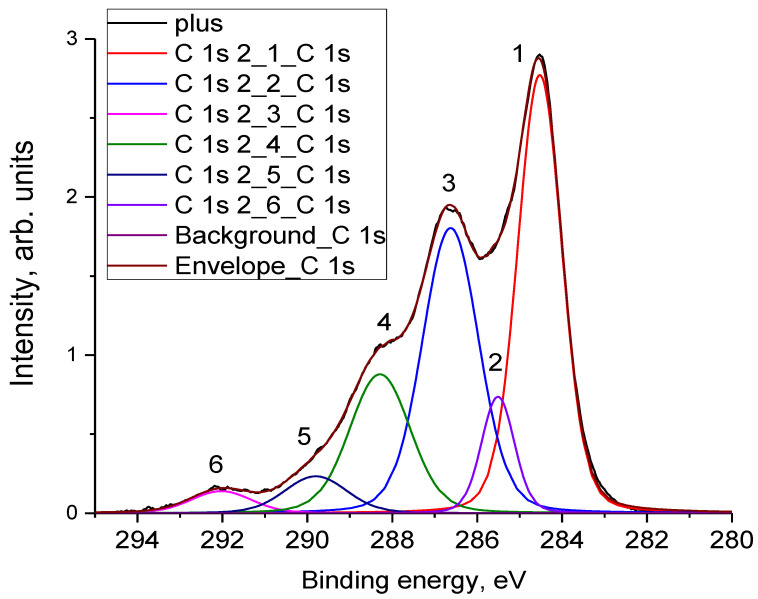
The C1*s* spectrum of the “positive” electrode and its decomposition.

The identification of oxygen-containing groups bound to the carbon atoms can be carried out by analyzing the C1*s* spectra. Figure 8 presents the high-energy resolution C1*s* spectrum of the “positive” electrode after background subtraction. The spectrum decomposition is shown in Table 2, where the assignment of individual peaks was performed in accordance with previous studies [37,38,39,40,41]. According to Figure 8 and Table 2, the main oxygen-containing functional groups in the studied samples are hydroxyl and epoxy groups, the concentration of which increases following the order initial < negative < positive. The concentration of carboxyl groups, which appear in the spectra as a peak with Eb = 288.0 eV, increases following the same sequence.

## 4. Discussion

Since the oxidation of the positive electrode should not lead to a significant increase in capacitance measured over the entire cell, the main contribution to an increase in the currents shown in Figure 5a can be considered to be due to the hydrophilization of both electrodes. This conjecture is supported by data obtained using the standard contact porosimetry [3]. According to these data, the original electrode has a part of the surface that is not wetted by water. The total specific surface area for original electrode equals to 1580 m^2^/g and it decreases to 940 m^2^/g for hydrophilic pores. The total porosity is 1.65 cm^3^/g; the porosity due to hydrophilic and hydrophobic pores is 1.31 and 0.34 cm^3^/g, respectively. The value of specific surface area of the carbon Norit is much higher compared to that of such materials such as, for example, lithium titanate [42,43].

The direct evidence of hydrophilization of both electrodes as a result of long-term polarization is provided by the change in the contact angle from 100° for the initial electrode to 50° for the negative electrode and complete wetting for the positive electrode.

It is known that oxygen-containing groups hydrophilize carbon materials. According to Table 2, the total content of such groups (C-OH, C-O-C and C(O)OH) is 47.66% and 45.48% for the positive and negative electrodes, respectively. Therefore, the positive electrode is more hydrophilized compared to the negative electrode, which is logical.

If hydrophilization of the positive electrode is a consequence of its electrochemical oxidation, then the mechanism of hydrophilization of the negative electrode is not clear at first glance. From the results of our IR and XPS measurements for the electrodes that underwent long-term polarization, the content of oxygen-containing functional groups on the electrode surfaces is higher than that of the initial electrode, regardless of polarity. Such groups are formed because of the chemical oxidation of carbon; that is, their formation is the result of self-discharge due to oxygen-containing radicals with a high oxidizing power. The formation of epoxy groups on the negative electrode should lead to its hydrophilization and an increase in capacity.

## 5. Conclusions

The results of this study can be formulated as follows.

(a) During the long-term stabilization (the potentiostatic charge), a decrease in the discharge capacity is observed at voltages exceeding 0.8 V; this is associated with the blocking of the surface by chemisorbed particles.
(b) The process of self-discharge can be conditionally divided into two stages: initial and main. For the initial stage, with a duration of about an hour, the voltage drop in the charged cell is determined by processes of charge redistribution inside the porous structure of the electrodes. For the main stage with a duration more than 2–3 h, the cell voltage drop is due to the processes of charge transfer between the electrodes.
(c) The self-discharge rate at the initial stage decreases with increasing stabilization time and is well-described by a simple first-order exponential equation over a wide time interval.
(d) The self-discharge rate of the main stage (~20 h) has a pronounced minimum. Under our experimental conditions, the optimal stabilization time of 50 min was obtained; this represented an increase of 60% in the residual energy compared to a cell charged without stabilization.
(e) According to the measurement of the contact (wetting) angle, hydrophilization of the negative electrode occurs during the long-term cell polarization process. In this case, the difference in the IR spectra of the negative electrode reveals the maximum corresponds to vibrations of the epoxy functional groups. Based on these data and considering that long-term stabilization leads to an increase in the average self-discharge current, an assumption was made about the participation of particles with a high oxidizing ability in the process of the main self-discharge.
(f) The oxidation of the electrodes that were in a charged state for a long time was established using XPS measurements. The oxidation state of the positive electrode significantly exceeds the oxidation state of the negative electrode, mainly due to the formation of hydroxyl and epoxy groups. The number of carboxyl groups on both electrodes increases to the same extent. The XPS data are consistent with the self-discharge mechanism 2 presented in the introduction.

## Figures and Tables

**Table 1:** Atomic concentrations (in %) in the initial electrode and the negative and positive electrodes after their testing.

Electrode	С	О	N	F	S
initial	81.27	11.98	2.54	4.05	-
negative	76.13	15.66	4	3.66	0.2
positive	74.81	16.83	4.25	3.75	0.19

**Table 2:** Decomposition of the C1*s* spectra of the electrodes under study.

Electrode	Peak	E_b_, eV	I, %	Assignment
Initial (0)	1	284.6	48.75	C–C (*sp*^2^)
	2	285.5	7.02	C–C (*sp*^3^)
	3	286.6	25.55	C–OH, C–O–C
	4	288.3	11.25	C(O)OH
	5	289.8	4.13	π→π*
	6	292.1	3.3	C–F_2_
Negative (−)	1	284.6	41.91	C–C (*sp*^2^)
	2	285.5	7.51	C–C (*sp*^3^)
	3	286.6	28.4	C–OH, C–O–C
	4	288.3	17.38	C(O)OH
	5	289.8	2.33	π→π*
	6	292.1	2.45	C–F_2_
	1	284.6	37.83	C–C (*sp*^2^)
	2	285.5	7.16	C–C (*sp*^3^)
Positive (+)	3	286.6	31.27	C–OH, C–O–C
	4	288.3	16.39	C(O)OH
	5	289.8	4.46	π→π*
	6	292.1	2.59	C–F_2_

π, π*—levels of multiple π-bonds.

## Data Availability

Not applicable.

## Supplementary Materials

The following supporting information can be downloaded at: https://www.mdpi.com/article/10.3390/ma16196415/s1, Figure S1: Photograph of a water drop on the electrode surface which was not subjected to polarization; Table S1: Photograph of a water drop on the electrode surface subjected to negative polarization. Video S1: A drop of water disappeared from the electrode surface.

## References

[1] Conway B.E. (2013). Electrochemical Supercapacitors: Scientific Fundamentals and Technological Applications.

[2] Bagotsky V.S., Skundin A.M., Volfkovich Y.M. (2015). Electrochemical Power Sources: Batteries, Fuel Cells, and Supercapacitors.

[3] Volfkovich Y.M., Bograchev D.A., Rychagov A.Y., Sosenkin V.E., Chaika M.Y. (2015). Supercapacitors with Carbon Electrodes. Energy Efficiency: Modeling and Experimental Verification. J. Solid State Electrochem..

[4] Oren Y. (2008). Capacitive Deionization (CDI) for Desalination and Water Treatment—Past, Present and Future (a Review). Desalination.

[5] Volfkovich Y.M. (2021). Electrochemical Supercapacitors (a Review). Russ. J. Electrochem..

[6] Kowal J., Avaroglu E., Chamekh F., Šenfelds A., Thien T., Wijaya D., Sauer D.U. (2011). Detailed Analysis of the Self-Discharge of Supercapacitors. J. Power Sources.

[7] Diab Y., Venet P., Gualous H., Rojat G. (2009). Self-Discharge Characterization and Modeling of Electrochemical Capacitor Used for Power Electronics Applications. IEEE Trans. Power Electron..

[8] Kurzweil P., Shamonin M. (2018). State-of-Charge Monitoring by Impedance Spectroscopy during Long-Term Self-Discharge of Supercapacitors and Lithium-Ion Batteries. Batteries.

[9] Liu K., Yu C., Guo W., Ni L., Yu J., Xie Y., Wang Z., Ren Y., Qiu J. (2021). Recent Research Advances of Self-Discharge in Supercapacitors: Mechanisms and Suppressing Strategies. J. Mater. Chem. A Mater. Energy Sustain..

[10] Tevi T., Yaghoubi H., Wang J., Takshi A. (2013). Application of Poly (p-Phenylene Oxide) as Blocking Layer to Reduce Self-Discharge in Supercapacitors. J. Power Sources.

[11] Shen J.-F., He Y.-J., Ma Z.-F. (2016). A Systematical Evaluation of Polynomial Based Equivalent Circuit Model for Charge Redistribution Dominated Self-Discharge Process in Supercapacitors. J. Power Sources.

[12] Saha P., Khanra M. Equivalent Circuit Model of Supercapacitor for Self-Discharge Analysis—A Comparative Study. Proceedings of the 2016 International Conference on Signal Processing, Communication, Power and Embedded System (SCOPES).

[13] El Brouji H., Vinassa J.-M., Briat O., Bertrand N., Woirgard E. Ultracapacitors Self Discharge Modelling Using a Physical Description of Porous Electrode Impedance. Proceedings of the 2008 IEEE Vehicle Power and Propulsion Conference.

[14] Bamgbopa M.O., Belaineh D., Mengistie D.A., Edberg J., Engquist I., Berggren M., Tybrandt K. (2021). Modelling of Heterogeneous Ion Transport in Conducting Polymer Supercapacitors. J. Mater. Chem. A Mater. Energy Sustain..

[15] Volfkovich Y.M., Rychagov A.Y., Mikhalin A.A., Sosenkin V.E., Kabachkov E.N., Shulga Y.M., Michtchenko A. (2022). Self-Discharge of a Supercapacitor with Electrodes Based on Activated Carbon Cloth. J. Electroanal. Chem..

[16] Wu F., Liu M., Li Y., Feng X., Zhang K., Bai Y., Wang X., Wu C. (2021). High-Mass-Loading Electrodes for Advanced Secondary Batteries and Supercapacitors. Electrochem. Energy Rev..

[17] Xu Z., Deng W., Wang X. (2021). 3D Hierarchical Carbon-Rich Micro-/Nanomaterials for Energy Storage and Catalysis. Electrochem. Energy Rev..

[18] Zhou J., Zhang S., Zhou Y.-N., Tang W., Yang J., Peng C., Guo Z. (2021). Biomass-Derived Carbon Materials for High-Performance Supercapacitors: Current Status and Perspective. Electrochem. Energy Rev..

[19] Wang X., Li X., Fan H., Ma L. (2022). Solid Electrolyte Interface in Zn-Based Battery Systems. Nano-Micro Lett..

[20] Li X., Wang X., Ma L., Huang W. (2022). Solvation Structures in Aqueous Metal-Ion Batteries. Adv. Energy Mater..

[21] Ke Q., Zhang Y., Fu Y., Yang C., Wu F., Li Z., Wei Y., Zhang K. (2023). Study on Electrochemical Performance of MnO@rGO/Carbon Fabric-Based Wearable Supercapacitors. Materials.

[22] Kumar S., Ahmed F., Shaalan N.M., Arshi N., Dalela S., Chae K.H. (2023). Investigations of Structural, Magnetic, and Electrochemical Properties of NiFe2O4 Nanoparticles as Electrode Materials for Supercapacitor Applications. Materials.

[23] Yang X., Fan H., Hu F., Chen S., Yan K., Ma L. (2023). Aqueous Zinc Batteries with Ultra-Fast Redox Kinetics and High Iodine Utilization Enabled by Iron Single Atom Catalysts. Nano-Micro Lett..

[24] Auer A., Ding X., Bandarenka A.S., Kunze-Liebhäuser J. (2021). The Potential of Zero Charge and the Electrochemical Interface Structure of Cu(111) in Alkaline Solutions. J. Phys. Chem. C Nanomater. Interfaces.

[25] Trasatti S., Lust E., White R.E., Bockris J.O., Conway B.E. (1999). The Potential of Zero Charge. Modern Aspects of Electrochemistry.

[26] Gajewska K., Moyseowicz A., Minta D., Gryglewicz G. (2023). Effect of Electrolyte and Carbon Material on the Electrochemical Performance of High-Voltage Aqueous Symmetric Supercapacitors. J. Mater. Sci..

[27] Klementov A.D., Litvinenko S.V., Stepanov A.B., Varakin I.N. Internal Losses and Features of Asymmetric Capacitor Operation. Proceedings of the 11th International Seminar on Double Lauer Capacitors and Similar Energy Storage Devices.

[28] Shulga Y.M., Baskakov S.A., Baskakova Y.V., Volfkovich Y.M., Shulga N.Y., Skryleva E.A., Parkhomenko Y.N., Belay K.G., Gutsev G.L., Rychagov A.Y. (2015). Supercapacitors with Graphene Oxide Separators and Reduced Graphite Oxide Electrodes. J. Power Sources.

[29] Si Y., Samulski E.T. (2008). Synthesis of Water Soluble Graphene. Nano Lett..

[30] Jeong H.-K., Lee Y.P., Jin M.H., Kim E.S., Bae J.J., Lee Y.H. (2009). Thermal Stability of Graphite Oxide. Chem. Phys. Lett..

[31] Cote L.J., Cruz− Silva R., Huang J. (2009). Flash Reduction and Patterning of Graphite Oxide and Its Polymer Composite. J. Am. Chem. Soc..

[32] Karthika P., Rajalakshmi N., Dhathathreyan K.S. (2012). Functionalized Graphene Oxide Based on Biomass Waste: Synthesis and Applications. Soft Nanosci. Lett..

[33] Xue Y., Liu J., Chen H., Wang R., Li D., Qu J., Dai L. (2012). Nitrogen-Doped Graphene Foams as Metal-Free Counter Electrodes in High-Performance Dye-Sensitized Solar Cells. Angew. Chem. Int. Ed Engl..

[34] Niu H., Zhang S., Wang R., Guo Z., Shang X., Gan W., Qin S., Wan L., Xu J. (2014). Dye-Sensitized Solar Cells Employing a Multifunctionalized Hierarchical SnO2 Nanoflower Structure Passivated by TiO_2_ Nanogranulum. J. Phys. Chem. C.

[35] Iamprasertkun P., Krittayavathananon A., Sawangphruk M. (2016). N-Doped Reduced Graphene Oxide Aerogel Coated on Carboxyl-Modified Carbon Fiber Paper for High-Performance Ionic-Liquid Supercapacitors. Carbon.

[36] Du C., Pan N. (2006). High Power Density Supercapacitor Electrodes of Carbon Nanotube Films by Electrophoretic Deposition. Nanotechnology.

[37] Arulepp M., Leis J., Lätt M., Miller F., Rumma K., Lust E., Burke A.F. (2006). The Advanced Carbide-Derived Carbon Based Supercapacitor. J. Power Sources.

[38] Park S., An J., Potts J.R., Velamakanni A., Murali S., Ruoff R.S. (2011). Hydrazine-Reduction of Graphite- and Graphene Oxide. Carbon.

[39] Shulga Y.M., Baskakov S.A., Knerelman E.I., Davidova G.I., Badamshina E.R., Shulga N.Y., Skryleva E.A., Agapov A.L., Voylov D.N., Sokolov A.P. (2013). Carbon Nanomaterial Produced by Microwave Exfoliation of Graphite Oxide: New Insights. RSC Adv..

[40] Voylov D.N., Agapov A.L., Sokolov A.P., Shulga Y.M., Arbuzov A.A. (2014). Room Temperature Reduction of Multilayer Graphene Oxide Film on a Copper Substrate: Penetration and Participation of Copper Phase in Redox Reactions. Carbon.

[41] Shulga Y.M., Baskakov S.A., Baskakova Y.V., Lobach A.S., Kabachkov E.N., Volfkovich Y.M., Sosenkin V.E., Shulga N.Y., Nefedkin S.I., Kumar Y. (2018). Preparation of Graphene Oxide-Humic Acid Composite-Based Ink for Printing Thin Film Electrodes for Micro-Supercapacitors. J. Alloys Compd..

[42] Pohjalainen E., Rauhala T., Valkeapää M., Kallioinen J., Kallio T. (2015). Effect of Li_4_Ti_5_O_12_ particle size on the performance of lithium ionbattery electrodes at high rates and low temperatures. Phys. Chem. C.

[43] Chaban M.O., Rozhdestvenska L.M., Palchyk O.V., Dzyazko Y.S., Dzyazko O.G. (2019). Structural characteristics and sorption properties of lithium-selective composite materials based on TiO_2_ and MnO_2_. Appl. Nanosci..

