# Supplementary material for: Self-Discharge Processes in Symmetrical Supercapacitors with Activated Carbon Electrodes
Source: Materials (Basel). 2023 Sep 26;16(19):6415. doi: 10.3390/ma16196415 (PMC10573834; doi:10.3390/ma16196415)
Supplement: Supplementary file 1 [file materials-16-06415-s001.zip › SI_figures.pdf]

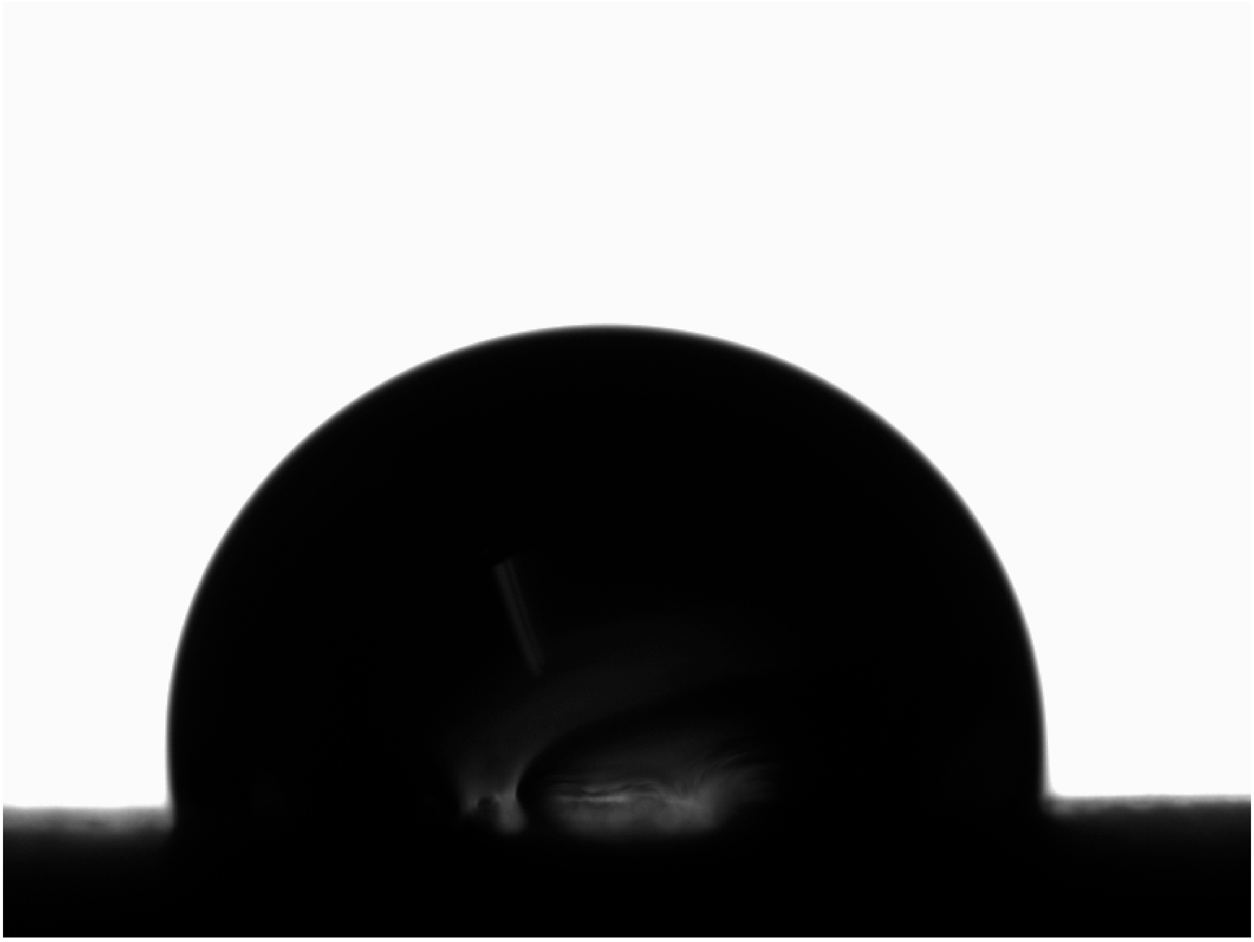

Figure S1. Photograph of a water drop on the electrode surface which was not subjected to polarization.

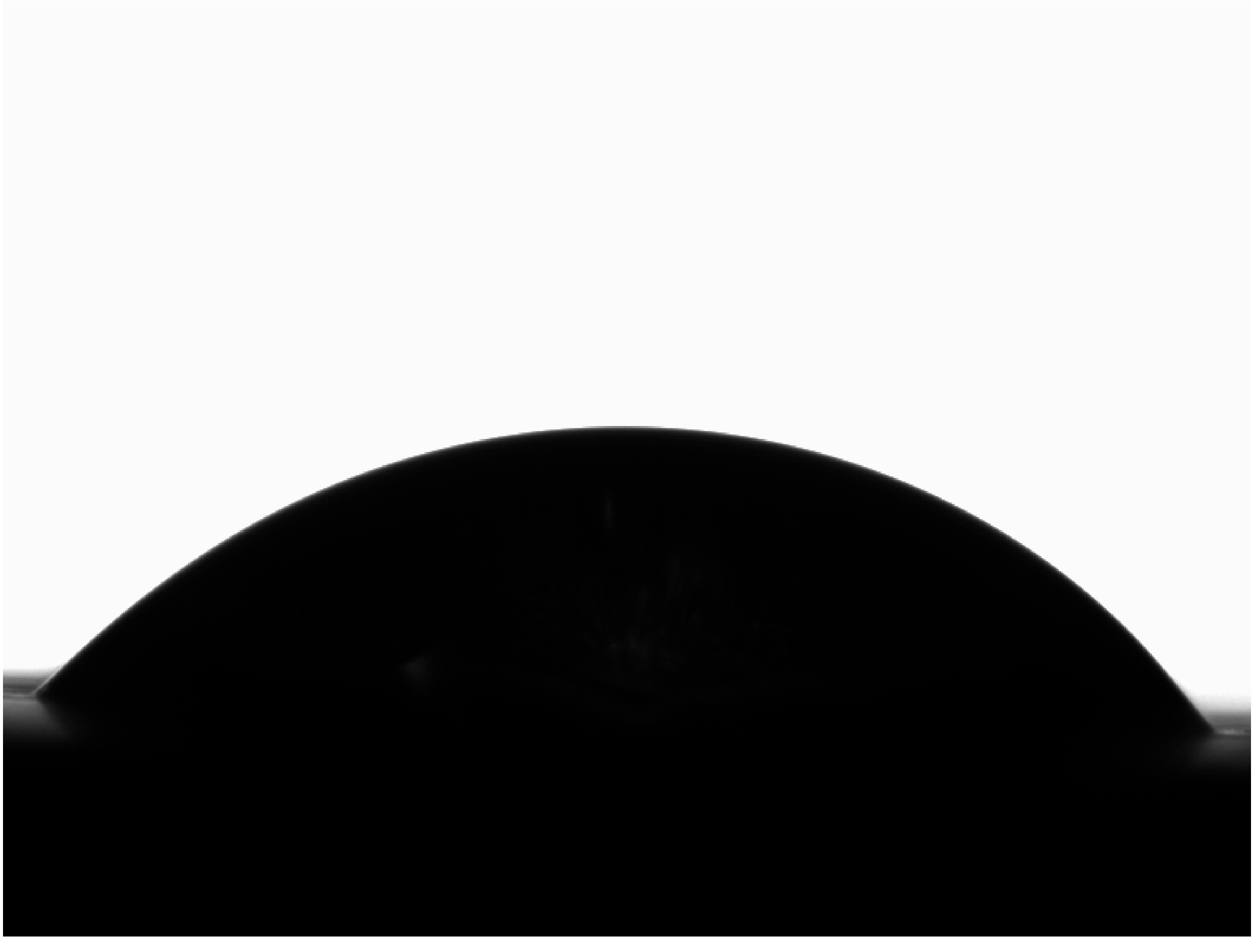

Figure S2. Photograph of a water drop on the electrode surface subjected to negative polarization.
